# Acute myocardial infarction following penetrating thoracic trauma: A case report and review of literature

**DOI:** 10.1016/j.ijscr.2019.11.022

**Published:** 2019-11-19

**Authors:** Sidra B. Bhuller, Sulman R. Hasan, John Weaver, Mark Lieser

**Affiliations:** a Department of Surgery, Sky Ridge Medical Center, 10101 RidgeGate Parkway, Lone Tree, CO 80124, USA; b Department of Internal Medicine, Oak Hill Hospital, 11375 Cortez Blvd, Brooksville, FL 34613, USA; c Department of Trauma Surgery, Research Medical Center, 2316 E Meyer Blvd, Kansas City, MO 64132, USA

**Keywords:** AMI, acute myocardial infarction, PTT, penetrating thoracic trauma, STEMI, ST elevation myocardial infarction, CAD, coronary artery disease, OR, operating room, TTE, transthoracic echocardiogram, LAD, left anterior descending, HTN, hypertension, SBP, systolic blood pressure, MTP, Massive transfusion protocol, pRBCs, packed red blood cells, FFP, fresh frozen plasma, LLL, left lower lobe, ASA, aspirin, BB, beta blocker, ARB, angiotensin II receptor blocker, SBTs, spontaneous breathing trials, HD, hospital day, BTT, blunt thoracic trauma, Penetrating thoracic trauma, Penetrating trauma, ST elevation myocardial infarction, Acute myocardial infarction

## Abstract

**Introduction:**

Acute myocardial infarction (AMI) as a result of penetrating thoracic trauma (PTT) is rare; however, there have been a few reports of AMI from gunshot wounds.

**Presentation of case:**

A patient without a history of coronary artery disease (CAD) presented with stab wounds to the left chest and underwent a left anterior thoracotomy, left lung wedge resection, negative pericardial window, and negative exploratory laparotomy. Shortly after leaving the operating room (OR) in a stable condition, the patient experienced a STEMI. An acute thrombus in the proximal left anterior descending (LAD) artery was identified.

**Discussion:**

AMI as a result of trauma, both blunt and penetrating, is extremely rare. There have been more reports of AMI secondary to blunt trauma, but it is much less common to find reports in literature of AMI from PTT.

**Conclusion:**

AMI as a result of penetrating trauma is rare, but can occur secondary to an acute thrombus, even in the absence of a direct cardiac injury. MI should be a consideration in patients with penetrating trauma to the chest, regardless of the intensity of the trauma. At minimum, a 12-lead electrocardiogram (ECG) and a baseline troponin should be obtained at initial evaluation and post-operatively, if surgically managed. If ECG demonstrates findings concerning for MI, it should be followed with an echocardiogram and/or cardiac angiogram to further help guide management, with an early cardiology consultation.

## Highlights

• AMI as a result of penetrating thoracic trauma is rare, but can occur secondary to an acute thrombus, even in the absence of a direct cardiac injury.
• Regardless of the cause of AMI, medical management should follow current guidelines.

## 1 Introduction

Acute myocardial infarction (AMI) as a result of penetrating thoracic trauma (PTT) is rare; however, there have been a few reports of MI from gunshot wounds [[1], [2], [3], [4], [5], [6], [7], [8], [9]]. Acute coronary thrombus with a ST elevation myocardial infarction (STEMI) from a stab wound, without a direct cardiac injury is extremely rare, especially in a young and otherwise healthy patient with no previous history of coronary artery disease (CAD). Medical literature is scarce in publications about AMI caused by PTT. Currently, there is no specific protocol on this issue. The work has been reported in line with the SCARE criteria [10].

## 2 Presentation of case

**Fig. 1:**
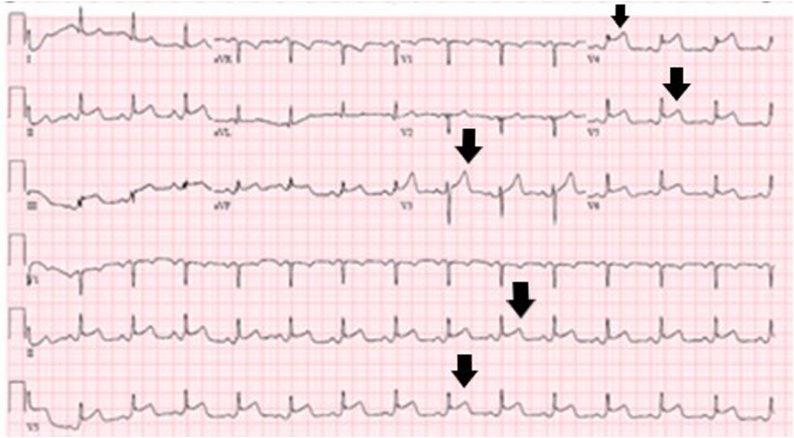
ECG with ST elevation myocardial infarction (STEMI) in the inferolateral leads (black arrows).

**Fig. 2:**
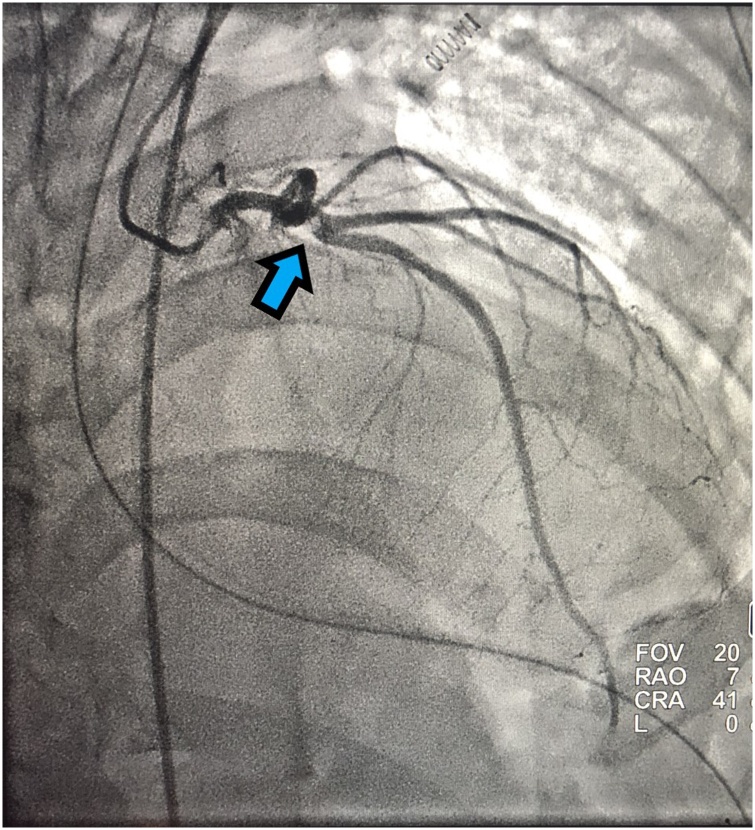
Acute thrombus in the proximal left anterior descending (LAD) artery (blue arrow).

**Fig. 3:**
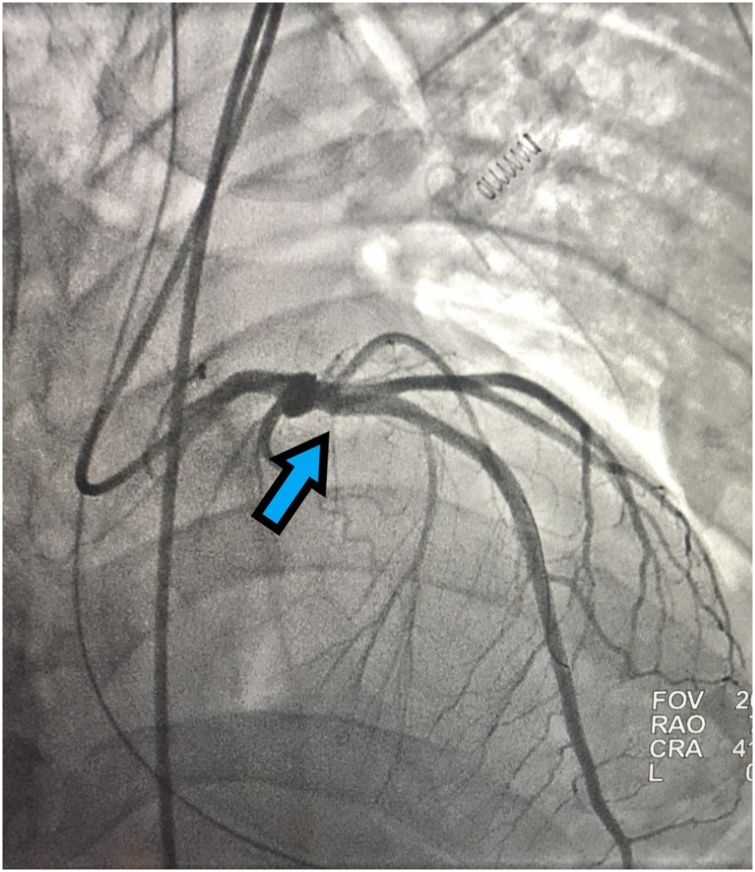
Resolution of acute thrombus after an aspiration thrombectomy was performed without significant residual narrowing (blue arrow).

A patient with a past medical history of hypertension (HTN), but without history of CAD, hyperlipidemia, or smoking presented with penetrating injuries to the left chest with systolic blood pressure (SBP) in the 80’s. Patient had diminished breath sounds on the left with low oxygen saturation in the trauma bay; a 32 French chest tube was inserted emergently with approximately 150 cc output of blood. Pt was also emergently intubated during this time for poor airway protection and low oxygen saturation. Massive transfusion protocol (MTP) was initiated; patient received two units of each packed red blood cells (pRBCs) and fresh frozen plasma (FFP) with stabilization of SBP into 130’s. Patient was noted to have stab wounds to the left anterior chest, left axilla, and posterior axillary line at the thoraco-abdominal junction. Patient subsequently underwent a left anterior thoracotomy, left lower lobe (LLL) lung wedge resection, a negative pericardial window, and a negative exploratory laparotomy. Lab work was grossly within normal limits without signs of hyper-coagulopathy. Shortly after leaving the operating room (OR) in a stable condition, patient experienced a STEMI in the inferolateral leads (Fig. 1) with elevated troponin. A transthoracic echocardiogram (TTE) was performed and showed an anteroapical wall motion abnormality; patient was taken emergently to the catheterization laboratory. Patient was found to have an acute thrombus in the proximal left anterior descending (LAD) artery (Fig. 2), and an aspiration thrombectomy was performed without significant residual narrowing (Fig. 3). No stent was placed since there was no clear evidence of CAD.

The cardiology team initiated a heparin drip, aspirin (ASA), beta blocker (BB), Aldactone, and angiotensin II receptor blocker (ARB) post thrombectomy for the management of acute STEMI. Patient was also started on Cardene drip for hypertension with SBP goal <150. Patient remained intubated secondary to failed daily spontaneous breathing trials (SBTs). On hospital day (HD) 3, patient was noted to be in hemorrhagic shock with hemoglobin (hgb) of 6.2 g/dL; of note, patient’s hgb on admission was 12.1 g/dL. After discussion with cardiology, a decision was made to stop the heparin drip and patient was transfused two units of pRBCs with an appropriate increase in hgb to 7.8 g/dL. Cardene drip was also discontinued given SBP in 90’s. A CT scan of the chest, abdomen, and pelvis was obtained with no obvious signs of active bleeding. On HD 4, patient self extubated and was alert and conversant. Patient’s hgb remained stable and deep venous thrombosis prophylaxis was initiated per trauma protocol on HD 5, and his hgb remained stable on HD 6. Patient had a repeat TTE which demonstrated normalization of previous anteroapical wall motion abnormality consistent with resolution of myocardial stunning on HD 7. Left chest tube was removed on HD 8. The patient was discharged home after 9 days in the hospital with no reported cardiac sequelae at discharge with low dose ASA, Plavix, BB, ARB, and Aldactone.

## 3 Discussion

AMI as a result of thoracic trauma, both blunt and penetrating, is extremely rare. There have been more reports of AMI secondary to blunt thoracic trauma (BTT), but it is less common to find reports in literature of AMI from PTT. Generally, coronary artery atherosclerosis is the most common cause of AMI; however, 20% of AMIs in young adults present secondary to non-atherosclerotic etiology; these include, but are not limited to coronary artery embolism, hypercoagulable state, coronary artery dissection, congenital coronary abnormalities, coronary artery spasms, vasculitis, and mediastinal irradiation. Trauma, both blunt and penetrating, is a less common underlying mechanism of AMI. Although rare, trauma patients can experience an AMI even without any commonly recognized risk factors of AMI; the pathophysiology is not well understood.

Although there is no specific protocol on this issue, the workup and management of AMI from a traumatic etiology should follow protocols for AMI from non-traumatic etiologies. A 12-lead EKG and serial troponins should be obtained. Based on the EKG findings, it should be followed by an echocardiogram. Cardiology should be consulted as early as possible. Based on the echocardiogram findings and cardiology evaluation, a cardiac arteriogram should be performed and patient should be taken to the catheterization laboratory if indicated. Medical management of AMI should follow current guidelines.

## 4 Conclusion

AMI as a result of PTT is rare, but can secondary to an acute thrombus, even in the absence of a direct cardiac injury. MI should be a consideration in patients with penetrating trauma to the chest. At minimum, a 12-lead electrocardiogram (ECG) should be obtained at initial evaluation and post-operatively, if surgically managed. If ECG demonstrates findings concerning for AMI, it should be followed with an echocardiogram and/or cardiac angiogram further help guide management, with an early cardiology consultation.

## Funding

None.

## Ethical approval

No ethical approval is required.

## Consent

Informed consent was unable to be obtained. The head of our medical team takes responsibility that exhaustive attempts have been made to contact the patient and that the paper has been sufficiently anonymized not to cause harm to the patient or their family. A signed document to this effect has been submitted.

## Author contribution

Sidra B. Bhuller DO, first author, contributed to the study concept, data collection, data analysis, and writing the paper; Sulman Hasan MD and John Weaver reviewed the manuscript; and Mark Lieser MD, senior author and the manuscript reviewer, contributed to the study concept, data analysis, and manuscript.

## Registration of research studies

None.

## Guarantor

Sidra B. Bhuller.

## Provenance and peer review

Not commissioned, externally peer-reviewed.

## Declaration of Competing Interest

None.
